# HMGB1 promotes Ox-LDL-induced endothelial cell damage by inhibiting PI3K/Akt signaling pathway

**DOI:** 10.1186/s12872-022-03003-y

**Published:** 2022-12-21

**Authors:** Xin Huo, Boyou Su, Guoti Qin, Liming Zhao

**Affiliations:** Department of Vascular Surgery, Liuzhou People’s Hospital, No. 8 Wenchang Road, Chengzhong District, Liuzhou, 545001 Guangxi China

**Keywords:** Atherosclerosis, High mobility group box-1, Oxidized low-density lipoprotein, PI3K/Akt pathway

## Abstract

**Background:**

Atherosclerosis is the pathological basis of cardio-cerebrovascular diseases. Oxidized low-density lipoprotein (ox-LDL) is an important risk factor for atherosclerosis. Ox-LDL leads to endothelial cell (EC) damage and dysfunction through various processes and promotes the occurrence and deterioration of atherosclerosis. High mobility group box-1 (HMGB1) is a protein associated with cellular damage. In the present study, the effect of HMGB1 on ox-LDL-induced EC damage was determined and the underlying mechanism explored.

**Materials and methods:**

Human umbilical vein ECs (HUVECs) were exposed to ox-LDL to induce endothelial damage and changes in HMGB1 expression level were detected using western blotting analysis and reverse transcription-quantitative PCR. To observe the effect of HMGB1 on ox-LDL-induced damage, the HMGB1 expression was downregulated with siRNA, and cell viability, cytotoxicity, and apoptosis rate were assessed. HUVECs were pretreated with LY294002, an inhibitor of the PI3K/Akt pathway, to determine whether the effect of HMGB1 on damage is via the PI3K-Akt pathway.

**Results:**

The results showed that ox-LDL can upregulate HMGB1 expression in HUVECs and downregulation of HMGB1 expression can prevent ox-LDL-induced damage in HUVECs. Furthermore, the effect of HMGB1 on ox-LDL-induced damage could be promoted by inhibiting the PI3K/Akt signaling pathway.

**Conclusion:**

The results indicate HMGB1 may be a promising research target to alleviate ox-LDL-induced EC damage.

**Supplementary Information:**

The online version contains supplementary material available at 10.1186/s12872-022-03003-y.

## Introduction

Atherosclerosis is the pathological basis of cardio-cerebrovascular diseases. Plaque attaches to arterial vessel walls and abnormal changes occur in the blood vessels. When plaque sloughs off and blocks a blood vessel, it causes ischemia or necrosis of the tissue or organ supplied by the artery, which can be fatal for the patient. Endothelial cells (ECs) are a critical line of defense for blood vessels against blood waste. There are multiple risk factors for EC damage, such as oxidized low-density lipoprotein (ox-LDL) [1], high glucose [2], and angiotensin II [3]. In pathological states, LDL is oxidatively modified to ox-LDL. Elevated ox-LDL levels predict acute cerebrovascular events [4], and are associated with poor functional outcomes after stroke [5]. Ox-LDL promotes the occurrence of atherosclerosis through multiple processes [1]. Thus, protection of ECs from ox-LDL damage is critical to prevent atherosclerosis.

Atherosclerosis is a progressive disease and patients should be closely monitored for changes in the levels of damaged factors. High mobility group box-1 (HMGB1) is a protein associated with cellular damage [6]. When cells are damaged by infection or other factors, cells rapidly release HMGB1. Extranuclear HMGB1 promotes inflammatory responses [7] and plays an important role in local or systemic chronic inflammatory diseases. During the formation of atherosclerosis, HMGB1 is abundantly expressed in ECs [8], vascular smooth muscle cells [9], and macrophages [10]. Therefore, HMGB expression is upregulated during the process from lipid deposition to fibrous plaque formation. Notably, high HMGB1 levels can also be detected in the serum and diseased tissues of patients with atherosclerosis-related diseases such as diabetes [11], ischemic stroke [12], and hypertension [13]. HMGB1 participates in the pathological process of atherosclerosis by binding to the receptor for advanced glycation end-product (RAGE) and toll-like receptors (TLRs) [9] indicating HMGB1 may be highly correlated with the occurrence and development of atherosclerosis.

In the present study, human umbilical vein ECs (HUVECs) were treated with ox-LDL to explore the relationship between HMGB1 and ox-LDL-induced EC damage. We hypothesized that HMGB1 plays a critical role in promoting ox-LDL-induced EC damage. HMGB1 mediates multiple signaling pathways such as PI3K/Akt, a key signaling pathway that regulates cell proliferation, differentiation, apoptosis, and senescence. When the PI3K/Akt pathway is activated, it regulates a variety of downstream effector molecules and participates in the occurrence and development of atherosclerosis. In the present study, changes in HMGB1 levels and Akt phosphorylation in damaged cells were examined.

## Materials and methods

### Cell culture and cell treatment

HUVECs were obtained from ATCC (Manassas, VA, USA). HUVECs were cultured in DMEM supplemented with 10% fetal bovine serum, 100 U/mL penicillin, and 100 µg/mL streptomycin and incubated in a humidified atmosphere with 5% CO_2_ at 37 °C. Ox-LDL was purchased from Peking Union-Biology Co. Ltd (Beijing, China). When cells reached 80% confluence, cells were trypsinized with 0.25% trypsin and passaged. To establish a cell model of ox-LDL-induced damage in HUVECs, concentration and time screening were performed. Briefly, different concentrations of ox-LDL (0, 25, 50, 100, or 150 μg/mL) were added to the HUVEC culture medium for 24 h and 100 μg/mL ox-LDL was added for 12 h, 24 h, or 48 h. To investigate the role of the PI3K/Akt pathway, HUVECs were pretreated for 1 h with the inhibitor LY294002 (10 μM, Sigma-Aldrich, Poole, UK).

### Cell transfection

To regulate the HMGB1 expression in HUVECs, small interfering RNA (siRNA) targeting HMGB1 (HMGB1-siRNA) and its siRNA negative control (NC-siRNA) were synthesized and obtained from Gene Pharma (Shanghai, China). Transfections were performed in 24-well plates using Lipofectamine 3000 (Invitrogen, Waltham, MA, USA) according to the manufacturer's instructions and assessed using western blotting.

### Cell viability and cytotoxicity assays

HUVECs were seeded in a 96-well plate at a density of 4 × 10^4^/well and the volume of each well was 100 μL. After LY294002 pretreatment and transfection, ox-LDL was added to the HUVECs medium and cultured for 24 h. Cell viability was detected using a CCK8 kit (Beyotime, Jiangsu, China) and cytotoxicity was detected using a LDH cytotoxicity assay kit (Beyotime). Briefly, after the cells were processed, the 96-well cell plate was removed, 10 µL of CCK8 solution was added to each well, and the plate was returned to the incubator for 1 h. The absorbance of each well was measured at 450 nm with a microplate reader (Bio-Rad, Hercules, CA, USA). When detecting cytotoxicity of HUVECs, the same cell treatment method described above was followed according to the manufacturer's instructions for the detection steps of the LDH kit; however, the absorbance of the sample was detected at 500 nm.

### Inflammatory factors detection

HUVECs were seeded in 6-well plates, and after incubation, the culture medium was collected and centrifuged at 500 g for 5 min to obtain the supernatant. The contents of inflammatory factors: tumor necrosis factor α (TNF-α), interleukin-1β (IL-1β), and IL-6 in the supernatant of HUVECs were detected by enzyme-linked immunosorbent assay (ELISA) kits (Beyotime). The operation steps are strictly in accordance with the manufacturer's instructions, and the absorbance of samples was read at 450 nm with the microplate reader.

### Annexin V/PI double-staining assay

HUVECs in logarithmic growth phase were seeded in a 6-well plate at a density of 1 × 10^6^/well. The next day, depending on the experiment, cells were sequentially transfected and LY294002 or ox-LDL was added to the cell culture medium. After the incubation step, the cell plate was removed and the medium carefully aspirated from each well. Cells in each well were trypsinized using trypsin without EDTA and the original medium was added to terminate the digestion. The cell suspension was centrifuged and the cell pellet washed with cold PBS solution. Next, 10 μL Annexin V-FITC and 5 μL PI were added to each tube and the reaction was performed at room temperature for 15 min in the dark. The apoptosis cells were detected using flow cytometry (BD Biosciences, San Jose, CA, USA).

### Western blot analysis

The HUVECs in each group were treated based on the experimental purpose, the cell plate was removed, and the cells washed with cold PBS solution. Experimental procedures were performed strictly according to the manufacturer's instructions. Total cell protein was extracted from RIPA lysate and the protein concentration was determined using the BCA method. Equal amounts of protein samples from each group were separated using SDS-PAGE electrophoresis and transferred to PVDF membranes (Bio-Rad). All blots were excised prior to hybridization with antibodies. PVDF membranes were blocked with 50 g/L skim milk followed by incubation overnight at 4 °C with primary antibodies: Cleaved Caspase-3 (1:1000, Cell Signaling Technology, Danvers, MA), Cleaved PARP (1:1000, Beyotime), anti-HMGB1 (1:1000, Abcam, Waltham, MA, USA), anti-Akt (1:1000, Beyotime), anti-phosphorylated Akt (p-Akt, 1:1000, Beyotime), anti-NF-κB p65 (1:1000, Boster, Wuhan, China), and GAPDH (1:1000, Beyotime). The next day, the membranes were incubated with horseradish peroxidase-conjugated secondary antibody (Boster) for 1 h at 37 °C. Then, ECL luminescent solution (Beyotime) was added to expose and develop labeled antibodies. The relative expression levels of HMGB1, Akt, and p-Akt proteins were analyzed using Image J software. The experiment was repeated three times.

### Reverse transcription (RT)-quantitative PCR

Total RNA of HUVECs in each group was extracted using the Trizol method. The total RNA was reverse transcribed into cDNA using a reverse transcription (RT) kit (TaKaRa, Beijing, China) in strict accordance with the manufacturer’s instructions. Quantitative PCR was used to amplify the cDNA using the SYBR Ex Taq kit (Takara Bio, Otsu, Japan). GAPDH served as the internal reference. The relative HMGB1 mRNA expression was calculated using the 2^−ΔΔCt^ method. The primer sequences used in this experiment are shown in Table 1.

**Table 1:** Sequences of RT-PCR primers

Gene name	Primer	Sequence 5′ > 3′
GAPDH	Forward	ACAGTTGCCATGTAGACC
	Reverse	TTTTTGGTTGAGCACAGG
HMGB1	Forward	AGCCCTCTTCATGTTCCGAAGTGT
	Reverse	TCATGTCAACACCTGCAGTCCCTT

### Immunofluorescence assay

8-mm cell slides were added to 48-well plates, and HUVECs were seeded in the plate for immunocytochemistry. After incubation, the plate was removed and the wells were washed 3 times with PBS for 5 min each. 75 μl of 4% paraformaldehyde was added to each well, fixed for 15 min at RT, and then the wells were rinsed 3 times with PBS. 50 μl of immunol staining blocking buffer was added to each well, incubated for 1 h at 37 °C, and then the wells were washed. Anti-HMGB1 (1:50, Beyotime) was added to each well for overnight incubation at 4 °C, the antibody solution was removed, and the wells were washed. The corresponding secondary antibody was added and incubated for 1 h at RT. Similarly, the secondary antibody solution was removed and the wells were washed. 100 μl of DAPI (0.5 μg/ml, Beyotime) was added to each well and incubated for 3 min at RT in the dark. 5 μl of anti-fluorescence quencher was dropped onto a glass slide, the slide was clipped out and placed upside down on the quencher, and the slide was sealed. After slides were completely dried, the slides were observed and photographed under a fluorescence microscope (ZEISS Microscopy, Jena, Germany).

### Statistical analysis

Data were expressed as the mean ± the standard error of the mean (SEM) and at least three independent experiments were performed for each result. Statistical analysis was performed using the GraphPad Prism 7.0 demo for Windows (GraphPad Software, San Diego, CA, USA). Differences between groups were analyzed using Student’s *t*-test or Mann–Whitney *U* test. A *p*-value < 0.05 was considered statistically significant.

## Results

### Establishment of an injury model of HUVECs induced by ox-LDL

**Fig. 1:**
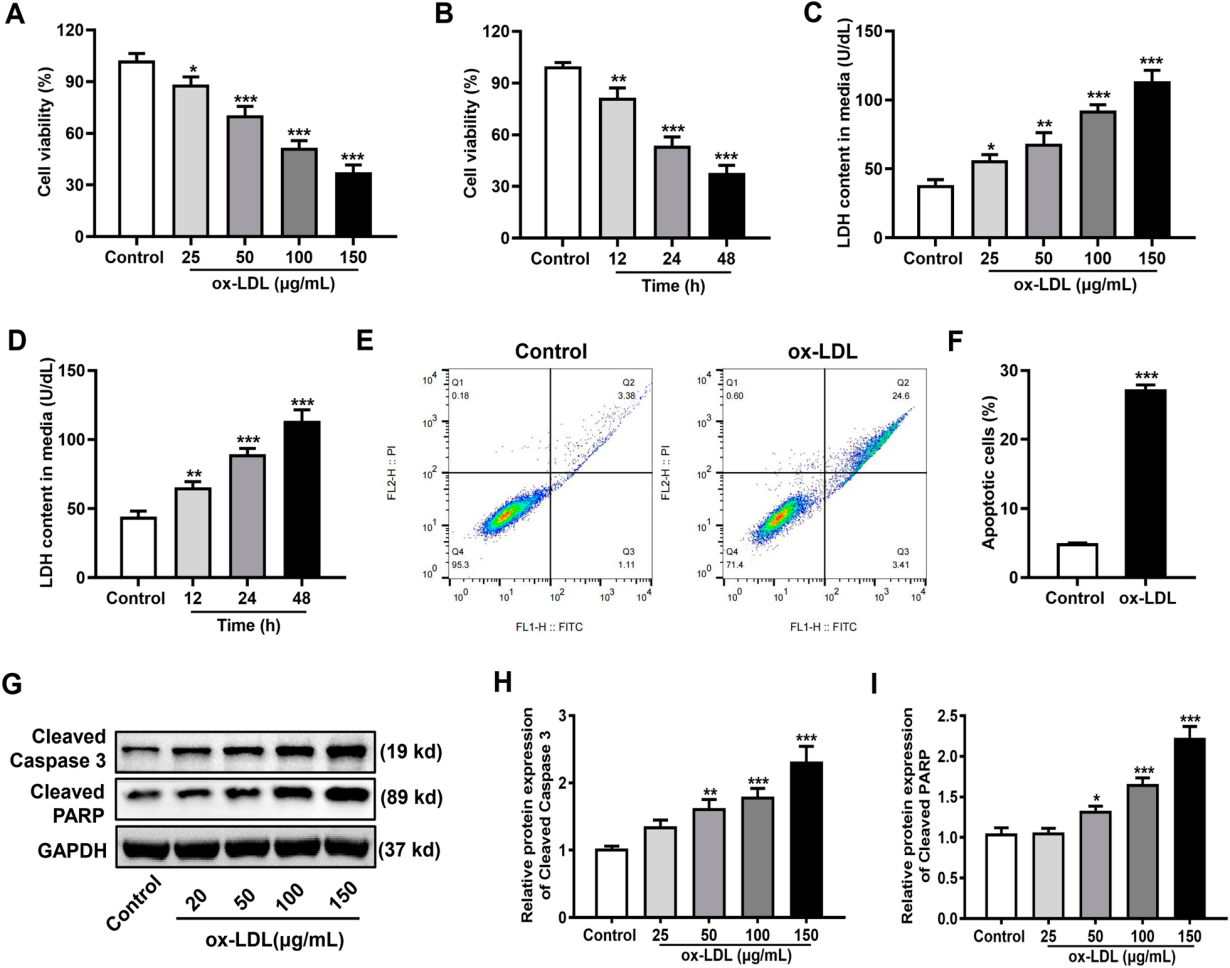
Changes in viability and apoptosis rate of HUVECs during ox-LDL-induced damage. **A** Cell viability after treatment with different concentrations of ox-LDL (25, 50, 100, or 150 μg/mL) detected using a CCK8 kit. **B** Cell viability at different time points (12, 24, or 48 h) when HUVECs were cultured with 100 μg/mL ox-LDL. **C** Cell cytotoxicity after treatment with different concentrations of ox-LDL detected using a LDH kit. **D** Cell cytotoxicity at different time points when HUVECs were cultured with 100 μg/mL ox-LDL. **E** Representative flow cytometry profiles of the control and ox-LDL groups. The HUVECs in the control group were untreated and the ox-LDL group was treated with 100 μg/mL ox-LDL for 24 h. **F** Percentage of apoptotic cells. **G** Representative western blot images of Cleaved Caspase-3 and Cleaved PARP expression. Original images of western blots are shown in Additional file 1: Supplementary Fig. 1A–C. The relative protein expression levels of **H** Cleaved Caspase-3 and **I** Cleaved PARP. Each experiment was independently repeated three times and the data were expressed as means ± SEM. ^*^p < 0.05, ^**^p < 0.01, and ^***^p < 0.001 versus Control

In the present study, cell viability in different groups was examined. As shown in Fig. 1A, B, the viability of HUVECs decreased in a concentration- and time-dependent manner. Furthermore, ox-LDL increased cytotoxicity and apoptosis rate of HUVECs. Based on the LDH release results (Fig. 1C, D), cytotoxicity also increased in a concentration- and time-dependent manner. Compared with the control group, the apoptosis rate of cells in the ox-LDL group (100 μg/mL) for 24 h increased (Fig. 1E, F). As the result shown in (Fig. 1G–I), the protein expression of apoptosis-related proteins, including Cleaved Caspase-3 and Cleaved PARP, was increased under treatment with different concentrations of ox-LDL (25, 50, 100, or 150 μg/mL). For the subsequent experiments, 100 μg/mL ox-LDL for 24 h was chosen to induce HUVEC damage. Overall, these data indicate that ox-LDL can induce HUVEC damage.

### Induction of ox-LDL increased HMGB1 expression

**Fig. 2:**
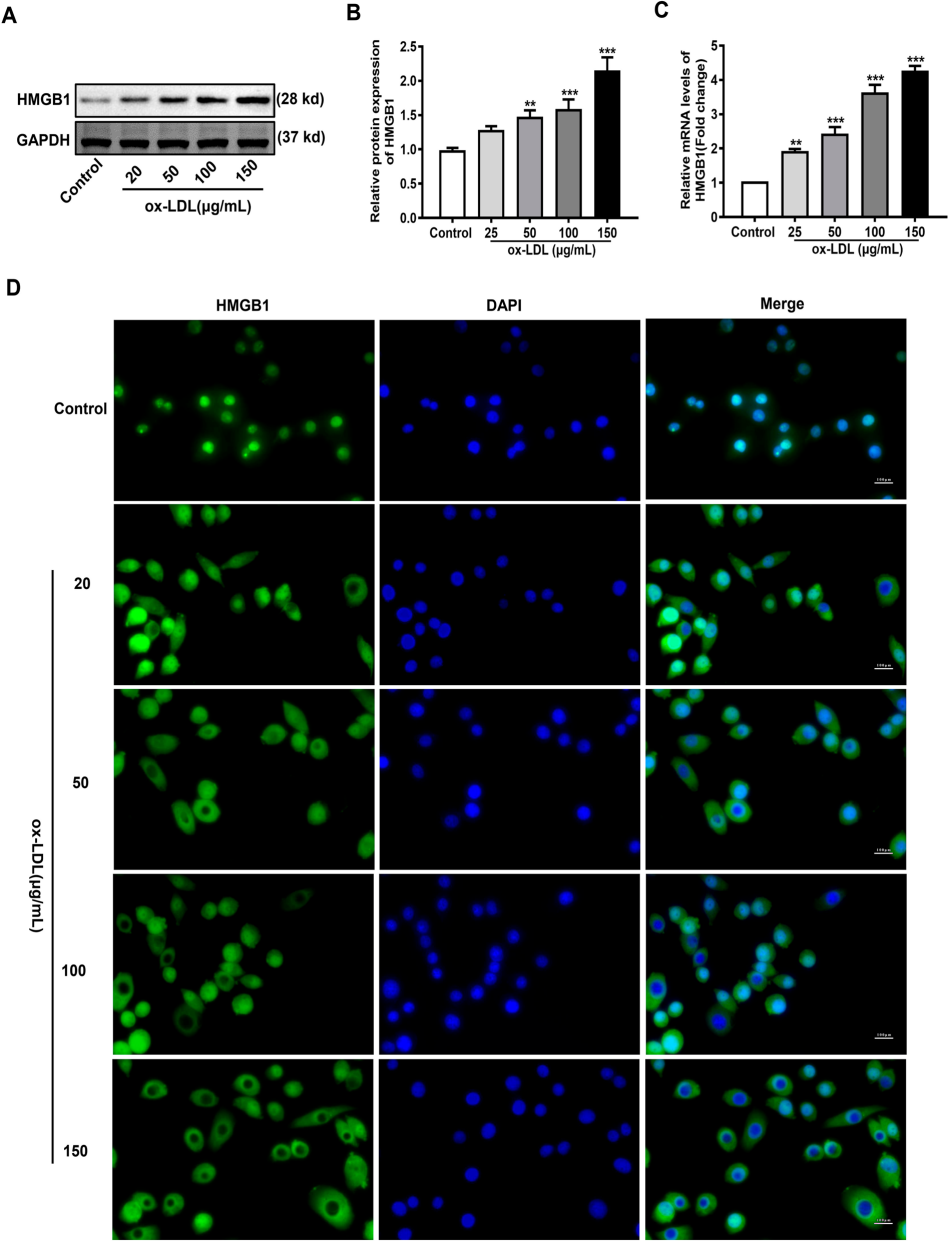
Induction of ox-LDL increases HMGB1 expression. **A** Representative western blotting images showing HMGB1 protein expression. GAPDH served as an internal control. Original images of western blots are shown in Additional file 1: Supplementary Fig. 1D, E. **B** The quantification of HMGB1/GAPDH protein ratio. **C** The HMGB1 mRMA expression level detected using RT-PCR. **D** Representative fluorescent images of HMGB1 (green) in each group (×400 magnification). The nuclei were stained by DAPI (blue). Scale bar = 100 nm. Each experiment was independently repeated three times and the data were expressed as means ± SEM. ^**^p < 0.01 and ^***^p < 0.001 versus Control

Whether ox-LDL could regulate the expression of HMGB1 in HUVECs was investigated. Changes in HMGB1 expression level in HUVECs was examined using western blotting, RT-PCR and immunofluorescence assay. As shown in Fig. 2A–C, compared with the control group, the protein and mRNA HMGB1 levels in the ox-LDL group were significantly increased. From immunofluorescence pictures (Fig. 2D), it was clear that positive staining of HMGB1 increased after ox-LDL induction, and the localization of HMGB1 was shifted from the nucleus to the cytoplasm. The above results indicate that ox-LDL induction can upregulate the expression of HMGB1 in HUVECs.

### Inhibition of HMGB1 expression attenuated ox-LDL-induced damage in HUVECs

**Fig. 3:**
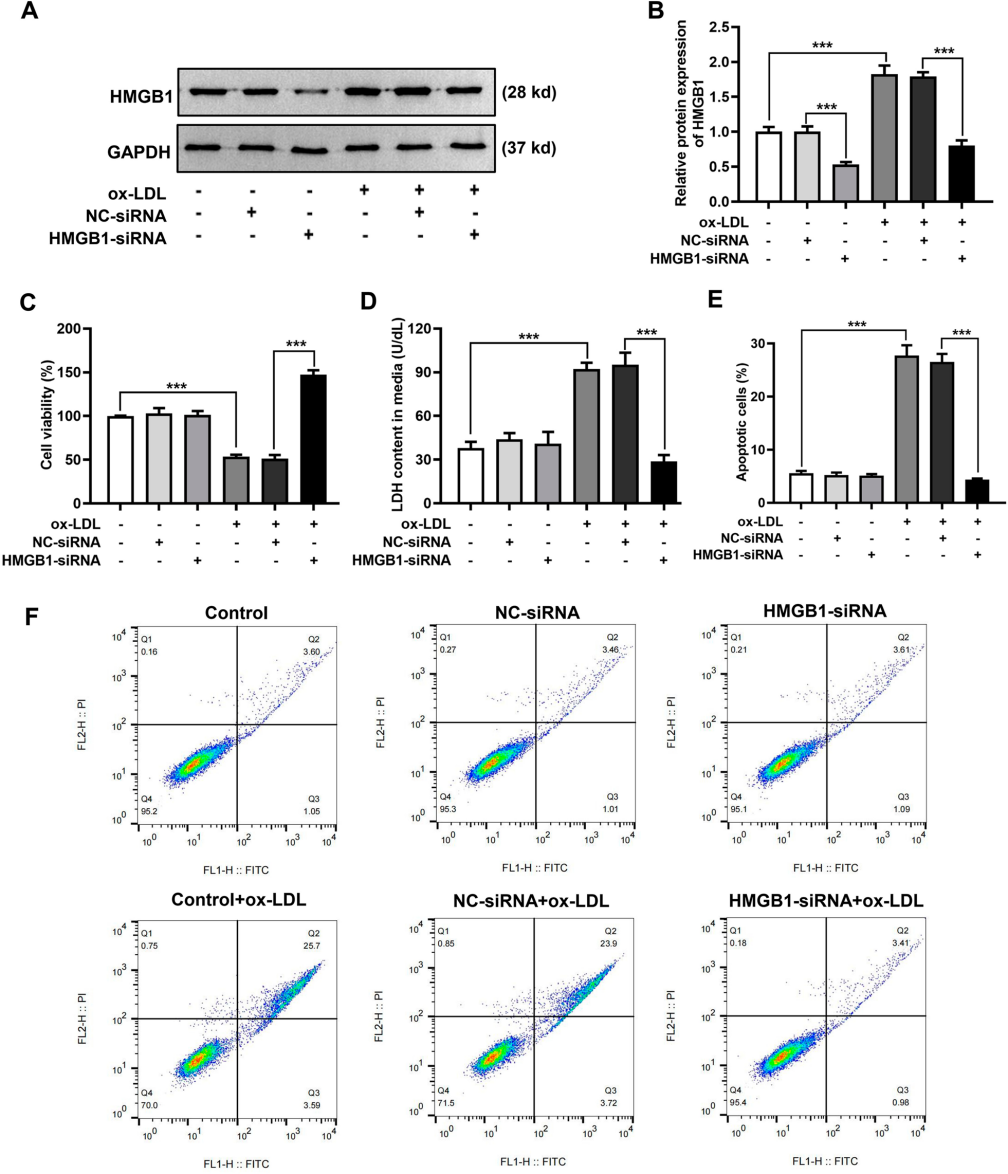
Inhibition of HMGB1 expression prevented ox-LDL-induced damage in HUVECs. **A** Representative protein bands of HMGB1. GAPDH served as a loading control. HMGB1-siRNA: small interfering RNA targeting HMGB1, NC-siRNA: small interfering RNA negative control. Original images of western blots are shown in Additional file 1: Supplementary Fig. 1F, G. **B** Quantitative analysis of (**A**) HMGB1 protein bands. **C** Cell viability in each group detected using the MTT assay. **D** Cell cytotoxicity detected using a LDH kit. **E** The percentage of apoptotic cells in each group in **F** was calculated. **F** Representative flow cytometry profiles of different treatment groups. ‘+’ was added, ‘–’ was blank. ^***^p < 0.001

To explore the relationship between HMGB1 and ox-LDL-induced damage, HMGB1 expression was downregulated with siRNAs. As shown in Fig. 3A, B, the HMGB1 protein level in the HMGB1-siRNA group was notably decreased compared with the NC-siRNA group. Similarly, the HMGB1 protein levels in the ox-LDL + HMGB1-siRNA group were decreased compared with the ox-LDL + NC-siRNA group. After HMGB1 downregulation, the viability, cytotoxicity, and apoptosis rate of HUVECs were altered. Compared with the ox-LDL + NC-siRNA group, the viability of HUVECs increased (Fig. 3C), LDH release decreased (Fig. 3D), and apoptosis rate decreased (Fig. 3E, F) in the group treated with ox-LDL and HMGB1-siRNA. In conclusion, downregulation of HMGB1 expression can prevent ox-LDL-induced damage in HUVECs.

### Inhibition of PI3K-Akt pathway promoted the role of HMGB1

**Fig. 4:**
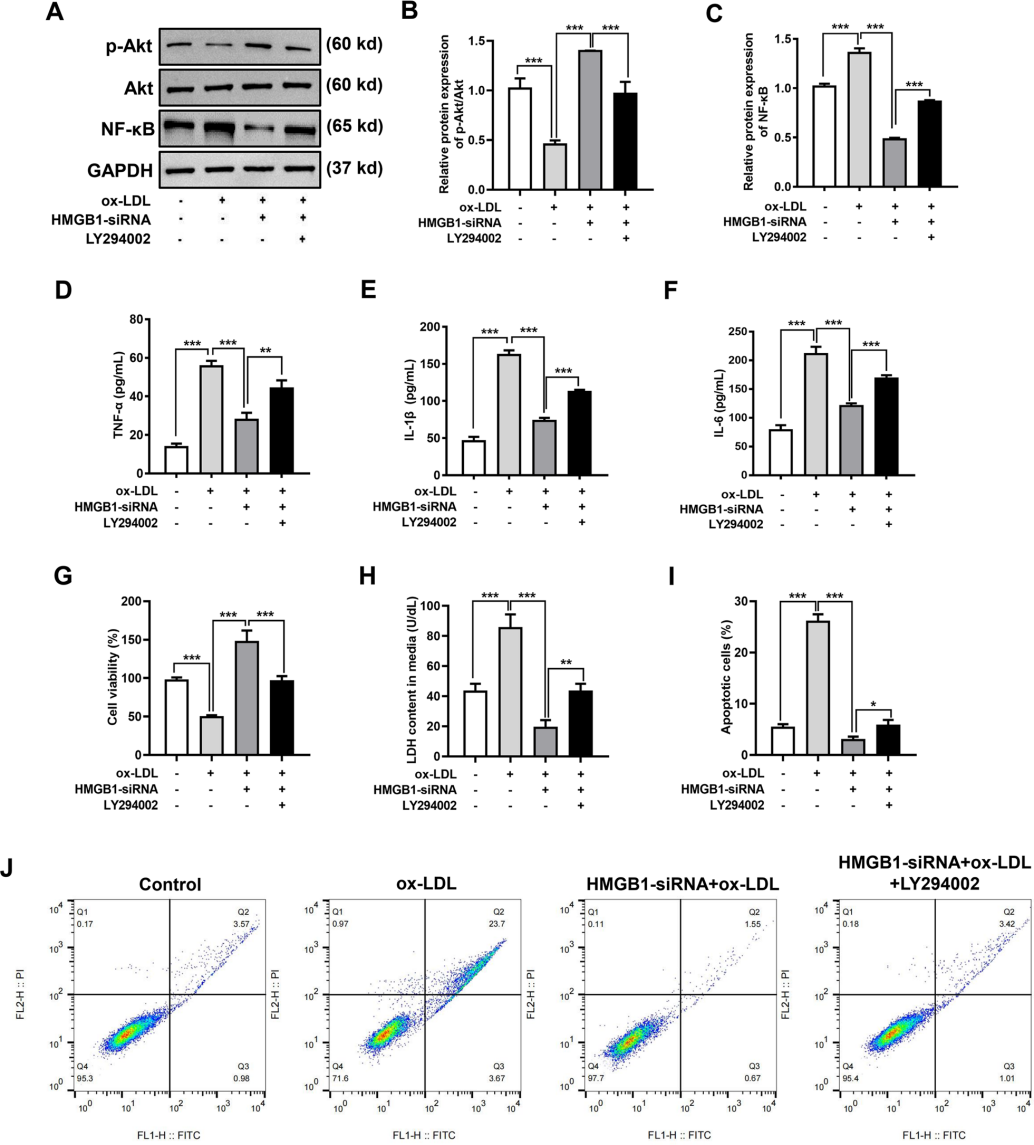
Inhibition of PI3K-Akt pathway promoted the role of HMGB1. HUVECs were pretreated with 10 μM LY294002 for 1 h. **A** Representative protein bands showing the expression of Akt, Akt phosphorylation, and NF-κB in HUVECs. GAPDH served as a loading control. Original images of western blots were shown in Additional file 1: Supplementary Fig. 1I–K. **B** The protein quantification ratio of P-Akt/Akt. **C** Relative protein expression levels of NF-κB. Quantitation of **D** TNF-α, **E** IL-1β, and **F** IL-6 in the supernatant of HUVECs was performed by ELISA. **G** Cell viability in each group detected using a CCK8 assay. **H** Cytotoxicity detected using a LDH kit. **I** The percentage of apoptotic cells in each group in **J** was calculated. **J** Representative flow cytometry profiles of different treatment groups. ‘+’ was added, ‘−‘ was blank. ^*^p < 0.05, ^**^p < 0.01, and ^***^p < 0.001

After ox-LDL induction, Akt phosphorylation was decreased (Fig. 4A, B), the expression level of NF-κB was increased (Fig. 4A, C), and the contents of downstream inflammatory factors TNF-α, IL-1β, and IL-6 were increased (Fig. 4D–F). To verify whether HMGB1 promotes damage through the PI3K-Akt pathway, cells were pretreated with the PI3K-Akt pathway inhibitor, LY294002. Compared with the ox-LDL + HMGB1-siRNA group, the Akt phosphorylation of HUVECs decreased, the expression level of NF-κB increased, inflammatory factors TNF-α, IL-1β, and IL-6 increased, cell viability decreased (Fig. 4G), LDH release increased (Fig. 4H), and apoptosis rate increased (Fig. 4I–J) in the ox-LDL + HMGB1-siRNA + LY294002 group. Based on the above results, the effect of HMGB1 on ox-LDL-induced damage of HUVECs could be promoted after inhibiting the PI3K-Akt signaling pathway.

## Discussion

ECs covering the lumen surface of blood vessels form a selective barrier between blood and blood vessels. In addition, ECs have endocrine functions such as the synthesis and release of a multitude of endothelial-derived vasoactive factors [14, 15]. In a previous study, EC damage and dysfunction were considered the early key contributors to the pathogenesis of atherosclerosis [16]. Initially, ECs from patients with atherosclerosis are chronically exposed to circulating inflammatory factors. EC injury is a potential response after prolonged exposure. Briefly, these changes trigger the disruption of the integrity of the endothelial monolayer, leading to the progressive formation of atheromatous plaques. Atherosclerosis mainly occurs in large and medium-sized arteries of the systemic circulatory system [17]. The pathological process is very long, causing stroke, myocardial infarction, and even sudden death [18].

Studies have shown that elevated ox-LDL levels can be detected in atherosclerotic plaques. Ox-LDL promotes foam cell formation [19, 20] and has cytotoxic effects that cause EC degeneration, necrosis, and shedding. The formation and rupture of atherosclerotic plaques are closely associated with ox‐LDL plasma levels [21]. Ox-LDL has predictive value for cardiac events in the general population and in patients with pre-existing cardiovascular disease [4]. In recent years, ox-LDL-induced injury of HUVECs is often used as a cellular model to study the pathogenesis of atherosclerosis [22]. In the present study, HUVECs were exposed to various concentrations of ox-LDL for different time periods. Consistent with other studies [22, 23], the ox-LDL group showed decreased cell viability, increased cytotoxicity, and increased apoptosis rate in a concentration- and time-dependent manner (Fig. 1). In addition, 100 µg/mL ox-LDL for 24 h was chosen as the optimal condition to induce damage in HUVECs. Our results showed the HMGB1 expression level in ECs increased after ox-LDL treatment, and HMGB1 could promote ox-LDL-induced endothelial damage by inhibiting the PI3K/Akt pathway.

HMGB1 is a highly conserved nuclear protein expressed in a variety of cells, and abnormally high HMGB1 expression can be found in lymphoid tissue, thymus, and tumor tissue. The biological role of HMGB1 is paradoxical and should be further investigated. In cardiovascular disease, cells are stimulated by pathogenic factors to release HMGB1, an inflammatory mediator. In the present study, whether ox-LDL affected the HMGB1 expression level in HUVECs was investigated. Consistent with previous findings [24], both the protein and mRNA HMGB1 levels were significantly increased in HUVECs exposed to ox-LDL (Fig. 2). Based on the results, HMGB1 may contribute to ox-LDL-induced injury in HUVECs. When HMGB1 expression was inhibited, the cell viability in the ox-LDL group was significantly increased and the cytotoxicity and apoptosis rate decreased (Fig. 3).

RAGE and TLRs are two canonical signaling pathways mediated by HMGB1. Recruiting inflammatory cells in the tissue injury area mediates inflammatory responses and promotes vascular injury by upregulating the expression of cytokines such as vascular adhesion molecules. The PI3K/Akt signaling pathway is associated with apoptosis. Reportedly, aristolochic acid induces apoptosis in HUVECs through the PI3K/Akt pathway [25].

The present study results showed the Akt phosphorylation expression level in the ox-LDL group was significantly lower than in the control group (Fig. 4A, B), indicating that ox-LDL could inhibit the Akt phosphorylation in HUVECs. To determine whether HMGB1 mediates the PI3K/Akt pathway, HUVECs were pretreated with LY294002. Compared with the ox-LDL + HMGB1-siRNA group, Akt phosphorylation was decreased in the ox-LDL + HMGB1-siRNA + LY294002 group. In addition, after blocking the PI3K/Akt signaling pathway, the protective effect of HMGB1-siRNA on EC injury disappeared. The results indicated the PI3K/Akt pathway may be involved in the regulation of HMGB1 in terms of EC damage. There are two major limitations in this study that could be addressed in future research. First, HMGB family proteins include HMGB1, HMGB2, and HMGB3. Although the company considered different isoforms when designing siRNA, the inhibitory effect of the siRNA on other isoforms cannot be completelv excluded. Second, the downstream effects of PI3K/Akt signaling pathway were not examined during ox-LDL induction. Although we detected the expression level of NF-κB and the content of inflammatory factors when HMGB1-siRNA or LY294002 was added.

## Conclusions

In conclusion, inhibiting HMGB1 expression can improve the viability of HUVECs and reduce apoptosis damage and its mechanism may be related to the activation of PI3K-Akt signaling pathway. Therefore, HMGB1 may be a promising research target to alleviate ox-LDL-induced EC damage.

## Supplementary Information

**Additional file 1:** Original images of western blots displayed in Fig. 1G, Fig. 2A, Fig. 3A and Fig. 4A.

## Data Availability

The datasets used and/or analysed during the current study available from the corresponding author on reasonable request.

## Abbreviations

ox-LDL: Oxidized low-density lipoprotein
HMGB1: High mobility group box-1
HUVECs: Human umbilical vein endothelial cells
ECs: Endothelial cells
RAGE: Receptor for advanced glycation end-product
TLRs: Toll-like receptors
si-RNA: Small interfering RNA
TNF-α: Tumor necrosis factor α
IL-1β: Interleukin-1β
IL-6: Interleukin-6
