# Supplementary material for: HMGB1 promotes Ox-LDL-induced endothelial cell damage by inhibiting PI3K/Akt signaling pathway
Source: BMC Cardiovasc Disord. 2022 Dec 21;22:555. doi: 10.1186/s12872-022-03003-y (PMC9768960; doi:10.1186/s12872-022-03003-y)
Supplement: Supplementary file 1 — Additional file 1: Original images of western blots displayed in Fig. 1G, Fig. 2A, Fig. 3A and Fig. 4A. [file 12872_2022_3003_MOESM1_ESM.docx]

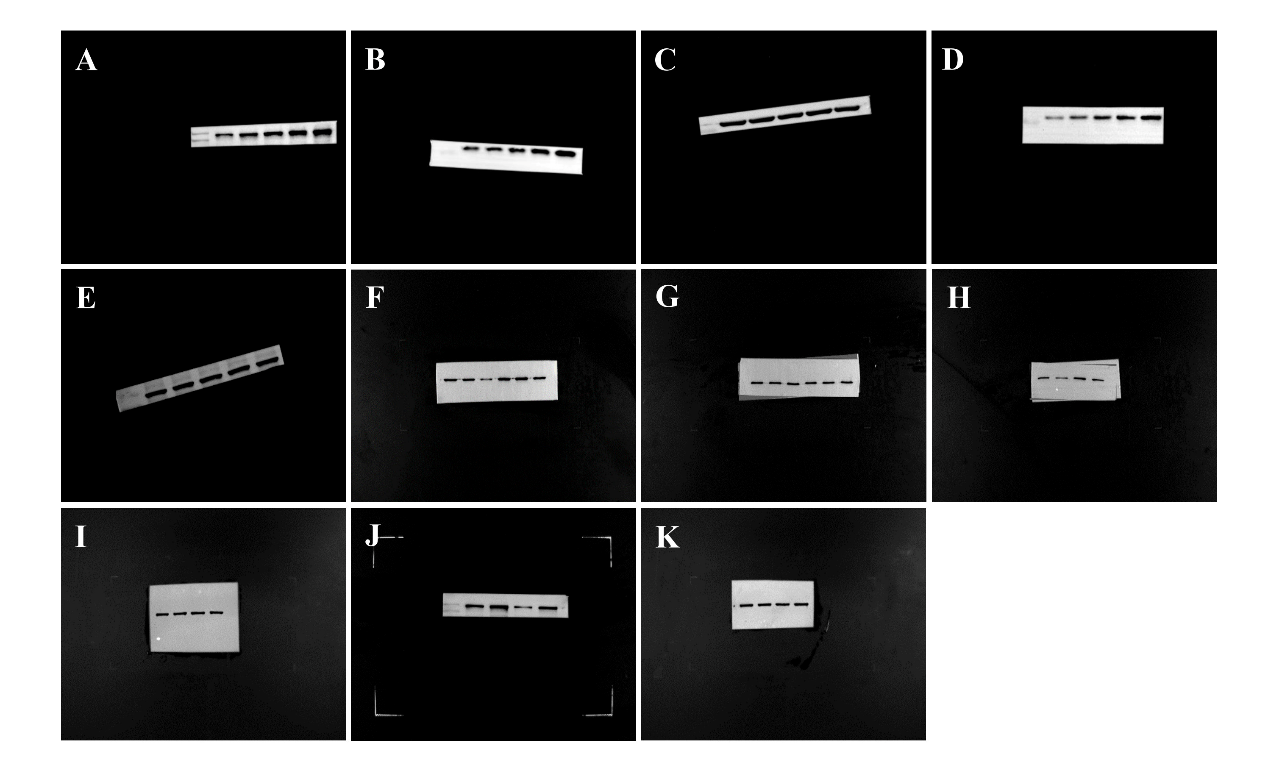


Supplementary Figure 1. Original images of blots. All blots were excised prior to hybridization with antibodies. Western blot images of (A) Cleaved Caspase-3, (B) Cleaved PARP, and (C) GAPDH in Figure 1G. Western blot images of (D) HMGB1 and (E) GAPDH in Figure 2A. Western blot images of (F) HMGB1 and (G) GAPDH in Figure 3A. Western blot images of (H) p-Akt, (I) Akt, (J) NF-κB, and (K) GAPDH in Figure 4A.
